# Thermochromic Smart Windows with Ultra-High Solar Modulation and Ultra-Fast Responsive Speed Based on Solid–Liquid Switchable Hydrogels

**DOI:** 10.34133/research.0462

**Published:** 2024-09-03

**Authors:** Guangjun Zhu, Gang Xu, Yu Zhang, Guo Lu, Xuan Cai, Wei Zhang, Wei She, Changwen Miao

**Affiliations:** ^1^ State Key Laboratory of High Performance Civil Engineering Materials, Southeast University, Nanjing 211189, China.; ^2^ School of Materials Science and Engineering, Southeast University, Nanjing 211189, China.; ^3^ Jiangsu Key Laboratory of Advanced Metallic Materials, Southeast University, Nanjing 211189, China.; ^4^ School of Civil Engineering and Architecture, Shandong University of Science and Technology, Qingdao 266590, China.; ^5^ Wuhan National Laboratory for Optoelectronics, School of Optical and Electronic Information, Huazhong University of Science and Technology, Wuhan 430074, China.

## Abstract

Thermo-responsive hydrogels can dynamically modulate incident light, providing a broad prospect for development of smart windows, which are of pivotal importance for energy conservation in buildings. However, these hydrogels normally exhibit slow response speed and tend to contract over extended phase transition, compromising structural integrity of smart windows. In this study, a solid–liquid switchable thermochromic hydrogel, denoted as SL-PNIPAm, was synthesized by cross-linking PNIPAm with AMEO through dynamic imine bonds. Due to its distinctive solid–liquid transformation characteristics, SL-PNIPAm demonstrates rapid response time (within 5 s) and retains structural integrity without undergoing shrinkage during heating/cooling and freezing/thawing cycles. SL-PNIPAm can also be encapsulated within 2 glass panels to prepare smart windows, which showed extraordinary luminous transmittance (*T*_lum_ = 96.8%) and solar modulation ability (Δ*T*_solar_ = 89.7%) and effectively reduced the indoor temperature (22 °C) in a simulated indoor experiment. Energy consumption simulation investigations are performed in diverse cities. The results reveal that SLW is capable of achieving a remarkable 54% reduction of HVAC energy consumption, leading to substantial decrease in CO_2_ emissions by up to 40 kg m^−2^ annually. This work develops a new hydrogel system with outstanding durability for smart windows and will promote the development and renovation of thermochromic smart windows.

## Introduction

In the world’s total energy consumption structure, fossil energy occupies a dominant position [1–3]. The emission of carbon dioxide (CO_2_) on a massive scale has resulted in substantial environmental impacts in terms of global warming [4,5], creating an urgent demand for energy conservation and emission reduction (ER) [6–8]. In 2022, the total CO_2_ emissions caused by energy consumption from building operations, which include heating, cooling, lighting, and equipment, reached a new high of 10 gigatons [9]. Windows are considered as the least energy-efficient part of the building envelope, during hot summers when strong sunlight can penetrate through them and generate excessive heat, leading to huge cooling demands. Even in winter, there is a huge heat loss.

Smart window [10,11] technologies (thermochromic [12–15], photochromic [16–18], electrochromic [19–21], etc.) have been developed to regulate the indoor temperature and brightness by reversibly modulating the transmission of sunlight, blocking or passing the solar irradiation according to the dynamic climate and personal preference. Thermochromic smart windows are considered as the most cost-effective and easy-to-implement strategy with zero energy input due to its ability to spontaneously adjust the transparent or opaque state to adapt to different ambient temperatures. Thermochromic hydrogels, such as hydroxypropyl cellulose (HPC) [22], poly (*N*-vinyl caprolactam) (PNVCL) [23], and poly (*N*-isopropyl acrylamide) (PNIPAm) [24–28], could experience reversible hydrophilic/hydrophobic phase transitions at lower critical solution temperature (LCST), resulting in the incident light to pass through at low temperature, while strongly scattering the incident light at high temperature, which make them ideal materials for smart windows.

PNIPAm hydrogels have attracted increasing attention due to the suitable LCST (32 °C) and excellent discoloration effect. However, the slow response speed limited the further development of thermochromic hydrogel smart windows. When the LCST is reached and phase separation occurs, free water is confined in the thermochromic hydrogel 3-dimensional (3D) network and flows slowly, resulting in a slow response speed (about 25 to 40 s). Increasing the pore size of PNIPAm hydrogel can accelerate the water flow in 3D hydrogel networks and subsequently reduce response time. For example, creating a second network with larger pore sizes within the hydrogel skeleton by either hydrogen bonding interaction [29] or physical entanglement [30] could markedly increase the aperture dimension due to the chain aggregate, resulting in the reduced response speeds (bidirectional transition) of 15 s. However, larger pore sizes make hydrogels more prone to water loss, reducing their structural integrity and temperature-responsive stability [31]. The obvious shrinkage phenomenon occurring after several heating/cooling or freezing/thawing cycles of thermochromic hydrogels is another limitation for their practical applications. The shrinkage of PNIPAm hydrogels makes the surface uneven, which not only reduces the transmittance and solar modulation as the light scattering effect increases but also prevents the complete coverage of the window. Despite efforts to augment the stability of the PNIPAm hydrogel’s 3D network through strategies such as introducing a secondary network layer [30] or incorporating monomers with enhanced strength [32], the occurrence of shrinkage persists. The application of PNIPAm polymer solution in the liquid state could overcome the shrinkage problem and fill every corner of the glass cavity [33]. Additionally, the liquid PNIPAm endows the smart window with an extremely fast response speed (5 s) since water can flow freely without being restricted by the 3D network. Nevertheless, liquid PNIPAm cannot fully block sunlight due to their weak sunlight scattering properties, especially in the infrared band, leading to increased cooling energy consumption.

Designing a PNIPAm system with solid–liquid transition properties that combine advantages of fast response speed, excellent cycle stability, and high solar modulation may provide an ideal solution. However, developing such system requires establishing temperature-responsive dynamic covalent bonds, which is challenging as most dynamic bonds can only be transformed at high temperatures (>100 °C) rather than at room temperature (around 30 °C) due to their high bond energy [34,35]. Although dynamic imine bonds can be transformed at room temperature, the intrinsic hydrolysis characteristic makes them decompose in water and difficult to function in hydrogel systems [36,37]. Fortunately, the molecular chains of PNIPAm network undergo phase separation from water at high temperatures, providing necessary conditions for imine bond formation. The physical crosslinking method also facilitates the transition of hydrogels between solid and liquid states [31], and it shows faster response speed and better durability.

**Fig. 1.**
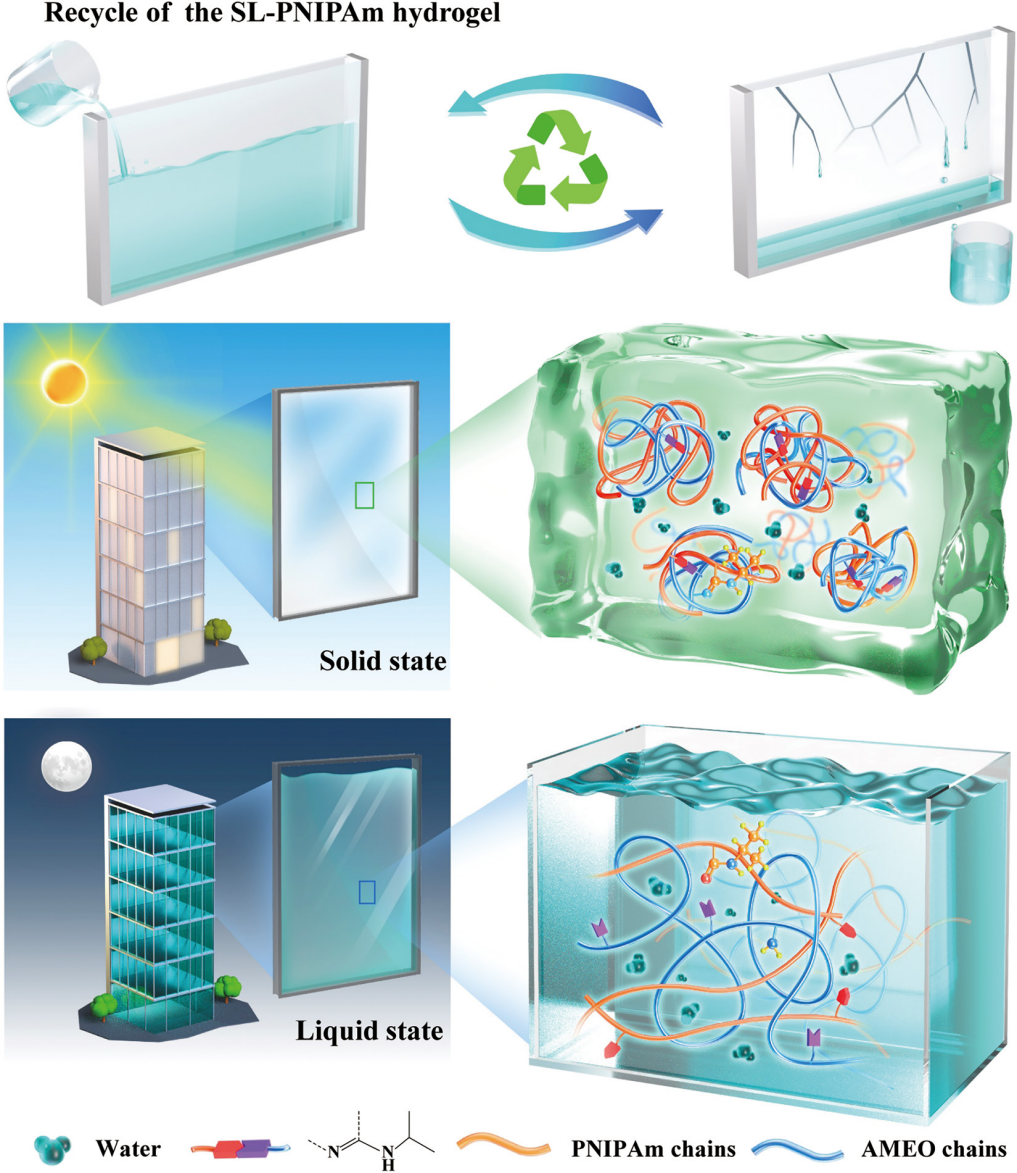
Concept and the design principle of the solid–liquid switchable hydrogel: Liquid SL-PNIPAm can be recycled when the window is aged or damaged. At high temperatures, the molecular chains of PNIPAm cross-link with the silanol network through imine bonds and form a 3D network, SL-PNIPAm is solid and opaque. At low temperatures, the PNIPAm molecular chain expands and no 3D network is formed, SL-PNIPAm is liquid and transparent.

In this work, we have developed a smart thermochromic system by cross-linking PNIPAm with silane coupling agent [3-aminopropyltriethoxysilane (AMEO)] via dynamic imine bonds. The breaking and formation of covalent bonds imbue the system with solid–liquid transition properties, as illustrated in Fig. 1. This enables a rapid switch (within 5 s) between the transparent (at low temperatures) and opaque (at high temperatures) states, effectively addressing the notable shrinkage issues observed in traditional thermochromic hydrogels. The solid–liquid transformed PNIPAm hydrogel (SL-PNIPAm) precursor solution could be easily filled in between 2 glass panels to form an SL-PNIPAm smart window (SLW) with excellent recycling performance (Fig. 1). SLW demonstrates excellent solar modulation ability (*T*_lum_ = 96.8%, Δ*T*_solar_ = 89.7%), and outdoor simulation experiments show that SLW substantially reduces indoor temperatures by 22 °C during summer. Simulation results show the excellent energy-saving effect in Beijing, Singapore, Phoenix, Rio de Janeiro, and Abu Dhabi, and SLW can save 54% of HVAC (heating, ventilation, and air-conditioning) energy consumption compared with normal double-layer glass windows while reducing CO_2_ emissions by 40 kg m^−2^ per year in Abu Dhabi. Furthermore, introducing dynamic covalent bonds into thermochromic smart windows for the first time allows for a perfect combination of the advantages of PNIPAm in both solid and liquid states. This advancement holds the potential to strongly promote the development and renovation of energy-efficient windows and greatly contribute to global building energy conservation in the low-carbon economy.

## Results and Discussion

**Fig. 2.**
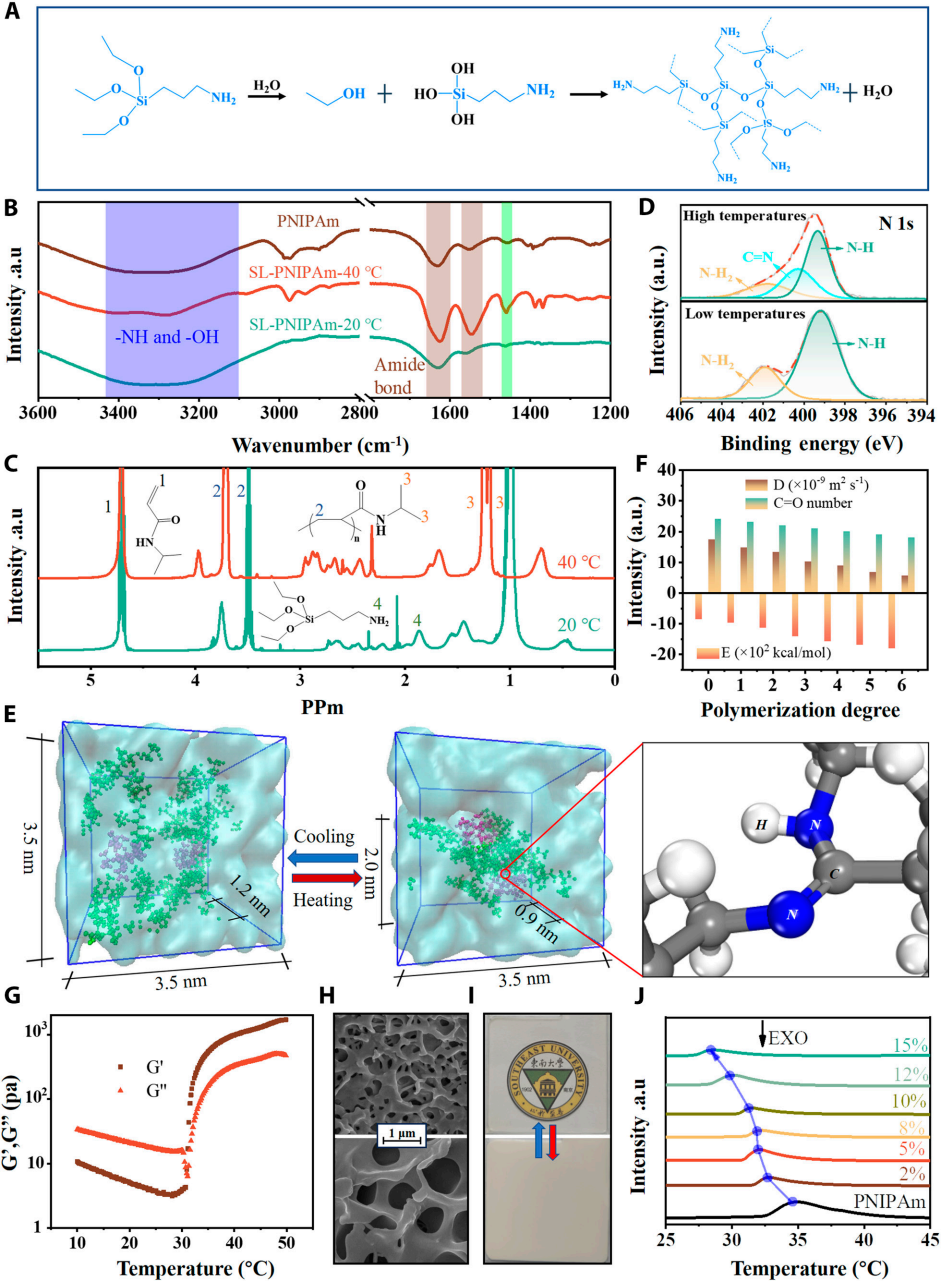
Characterization and molecular dynamic simulations of SL-PNIPAm. (A) AMEO undergoes hydrolysis to form silanol and then reacts to create a macromolecule crosslinking agent (MCA), with the dashed lines representing the undrawn chemical structures. (B) FT-IR spectra of SL-PNIPAm and pristine PNIPAm hydrogel. (C) NMR hydrogen spectra of SL-PNIPAm at different temperatures. (D) XPS survey spectra of SL-PNIPAm (N1s). (E) Molecular dynamic simulations of SL-PNIPAm at high and low temperatures. (F) Changes in energy, diffusion coefficient, and C═O content of SL-PNIPAm with increasing degree of polymerization. (G) Rheological behavior of SL-PNIPAm hydrogels at different temperatures. (H) SEM of freeze-dried SL-PNIPAm at 20 and 40 °C. (I) Photos of a 5 × 5 cm^2^ window response within 5 s. (J) LCST of SL-PNIPAm decreased with the increasing AMEO.

In contrast to the fixed crosslinking points of pristine PNIPAm, SL-PNIPAm was synthesized via dynamically switchable crosslinking of AMEO to realize the solid–liquid transition. In water, H_2_N-(CH_2_)_3_-Si-(O-C_2_H_5_)_3_ undergoes hydrolysis to form silanol (-Si-OH), which will subsequently create a macromolecule crosslinking agent (MCA) depicted in Fig. 2A. At low temperatures, PNIPAm fails to form a 3D network because MCA cannot form imine bond with the C═O bond of PNIPAm in water-rich environments, resulting in a fluid liquid state of SL-PNIPAm. As the temperature rises, the molecular chains of PNIPAm undergo a transition from the expanded state to the contracted state and phase separation occurs, which provides the ideal conditions for the formation of imine bond with the silanol network, resulting in a crosslinked 3D SL-PNIPAm network in a solid state (Fig. S1). The Fourier transform infrared (FT-IR) spectrum of both SL-PNIPAm and pristine PNIPAm hydrogels is presented in Fig. 2B. The typical characteristic peaks can be found at 1,630 cm^−1^ (C═O stretching) and 1,545 cm^−1^ (N–H bending), assigned to the amide I and II bands, respectively. The broad peak at 3,347 cm^−1^ corresponds to O–H stretching from structured water and N–H stretching from -NH_2_ groups. The nuclear magnetic resonance hydrogen spectra (^1^H NMR) of SL-PNIPAm at different temperatures are shown in Fig. 2C. The chemical shift at 1.85 parts per million (ppm) was attributed to the presence of NH_2_ at low temperatures. As the temperature rises, the characteristic response of NH_2_ disappears when SL-PNIPAm transforms into a solid state due to the formation of imine bonds between NH_2_ and C═O, resulting in a marked decrease in the amount of hydrogen atoms in this chemical environment. The peak at 4.75 ppm was attributed to the ═CH_2_ from unreacted *N*-isopropylacrylamide (NIPAm) monomer. It is important to note that temperature-induced phase separation in the presence of deuteroxide alters the solvent environment of the PNIPAm molecular chain, leading to changes in the chemical shifts of -CH_3_ and -CH_2_-. X-ray photoelectron spectroscopy (XPS) analysis further verified the formation of imine bonds [38,39] in SL-PNIPAm at elevated temperatures. The N1s spectrum in Fig. S2 illustrates that at lower temperatures, peaks at 399.2 and 401.9 eV correspond to NH and NH_2_, respectively. As temperature rises, the NH_2_ peak diminishes, concurrently with the emergence of the C═N (imine bond) peak at 400.2 eV, indicative of NH_2_ transforming into imine bonds.

Molecular dynamic simulations were employed to explore the distinct states of SL-PNIPAm, as depicted in Fig. 2D. At lower temperatures, the PNIPAm chains (green) and MCA (purple) are distributed within the liquid, and PNIPAm chains undergo contraction as the temperature rises. This structural evolution during temperature elevation is driven by the consumption of C═O bonds in PNIPAm and the subsequent formation of imine bonds between PNIPAm chains and MCA. As demonstrated in Fig. 2D, the reduction of C═O bonds with the polymerization degree leads to a decrease in the hydrophilicity of the polymer system, causing water molecules to be expelled to a greater extent (Fig. 2E). Additionally, the formation of a 3D network greatly hinders the mobility of polymer molecules. The self-diffusion coefficient of carbon in the polymer decreases from 17 × 10^−9^ m^2^/s to 4 × 10^−9^ m^2^/s, with the polymerization degree ranging from 1 to 6, indicating a gradual transition from a liquid state to a solid state. This process facilitates the evolution, specifically the phase separation, of the polymer with water and the phase change of the polymer. The overall process is advantageous in terms of energy, as evidenced by a decrease in the potential energy of the composite system from −8.6 × 10^2^ kcal/mol to −18 × 10^2^ kcal/mol as the polymerization degree decreases from 1 to 6.

The rheological behavior of SL-PNIPAm hydrogels around the LCST was investigated (Fig. 2G) by heating the samples from 10 to 50 °C. The SLW hydrogel exhibited abrupt changes in both storage modulus (*G*′) and loss modulus (*G*″) with increasing temperature. At low temperatures, *G*′ of SL-PNIPAm is lower than *G*″, indicating a viscous behavior where SL-PNIPAm flows freely like a liquid. Upon reaching the LCST, *G*′ surpasses *G*″, demonstrating elastic behavior. At this state, SL-PNIPAm becomes opaque and gains mechanical properties, behaving like a solid. Therefore, SL-PNIPAm can dynamically switch between solid and liquid states with temperature changes. Unmodified PNIPAm (without AMEO) lacks the phase transition behavior observed in SL-PNIPAm. At low temperatures, *G*′ and *G*″ are comparable, indicating a semisolid state. As temperature rises, *G*′ surpasses *G*″, leading to solidification, accompanied by irreversible volume shrinkage and water loss (Fig. S2). Since no crosslinking agent is added, unmodified PNIPAm lacks a 3D network, causing its molecular chains to contract and cluster upon heating, resulting in irreversible volume shrinkage and water loss. The results of gel chromatography showed that the weight average molecular weight of SL-PNIPAm was about 234,752 (Fig. S3), which proved that SL-PNIPAm was a solution of PNIPAm molecular chains at low temperature, so it was liquid.

To analyze the microstructure of SL-PNIPAm, samples were placed at 20 and 40 °C for 1 h, rapidly frozen with liquid nitrogen, and then freeze-dried to preserve their structure (Fig. 2H). At 20 °C, SL-PNIPAm exhibits relatively small pore sizes due to the physical cross-linking of PNIPAm chains [31], resulting in a porous structure after freeze-drying. As the temperature increases to 40 °C, hydrophobic interactions between the PNIPAm chains strengthen and SL-PNIPAm cross-links with the silanol network via imine bonds. A substantial increase in pore size is observed. This phenomenon is attributed to the contraction of PNIPAm molecular chains at high temperatures, which enhances the scattering effect of the shrinking and agglomerating chains on sunlight, resulting in the transition from transparent to opaque SL-PNIPAm. Figure S4 displays the scanning electron microscopy (SEM) image of pristine PNIPAm hydrogel at various temperatures, showing a similar trend to SL-PNIPAm.

Response time is one of the key performance indexes for smart window, determining the switching rate between the transparent and opaque states of the thermochromic window. A fast response speed allows the thermochromic window to quickly adapt to changes in external temperature, thereby enhancing energy efficiency and improving indoor comfort. Figure S6A illustrates the response time of SL-PNIPAm and pristine PNIPAm hydrogel for a complete transition. During the heating process from 20 to 40 °C, SL-PNIPAm transitions from transparent to completely opaque within 5 s (Fig. 2I and Movie S1). Similarly, during the cooling process, SL-PNIPAm also responds within 5 s. The transmittance time curve at 650 nm (Fig. S6B) provides a clearer depiction of the response speed of SL-PNIPAm, which can respond within 5 s, whether heated or cooled. Due to its rapid response capabilities, SL-PNIPAm exhibits promising potential for application in the domains of color display and optics [29]. In contrast, pristine PNIPAm takes 23 s to transition from transparent to opaque and 15 s from opaque to transparent. At 20 °C, SL-PNIPAm is in a flowing liquid state that facilitates the unimpeded flow of water, contributing to its fast response. Upon reaching 40 °C, as SL-PNIPAm undergoes the transition from a liquid state to a solid state, the process of achieving transparency involves a concurrent transformation into a liquid state, enhancing its response speed. We further examined the effect of AMEO content on the LCST of SL-PNIPAm. As shown in Fig. 2J, the LCST decreased from 32 to 27 °C with the increase of AMEO concentration, which is beneficial for SLW to adapt to different climatic regions. The oxygen and nitrogen atoms in AMEO could form hydrogen bonds with water, resulting in reduced hydrogen bonds between PNIPAm molecular chains and water, consequently reducing the LCST of SL-PNIPAm. It is noted that SL-PNIPAm containing 5% AMEO was employed in all subsequent tests due to its suitable LCST of 30.5 °C.

**Fig. 3.**
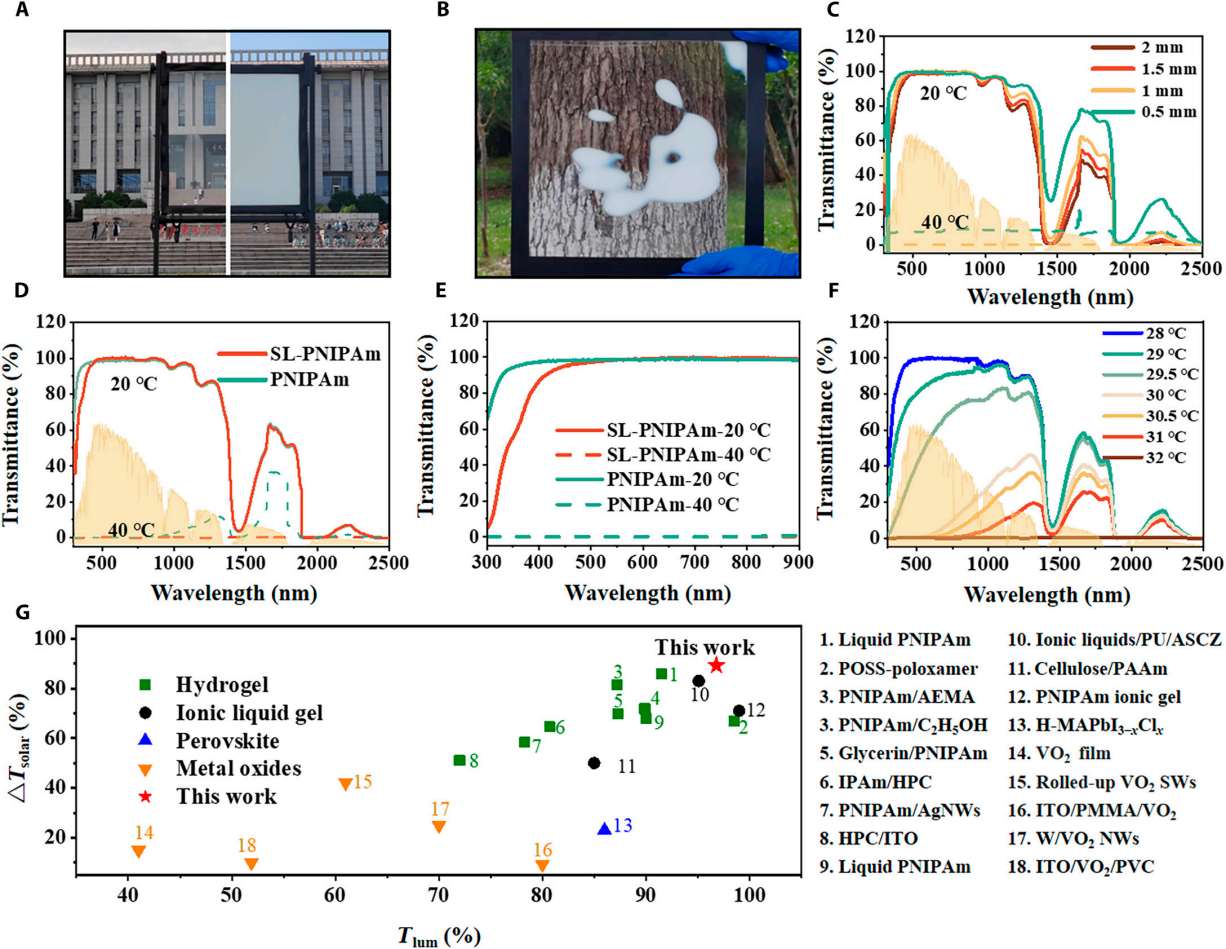
Thermochromic performance of SLW. Photo of a 50 × 50 cm^2^ window changing transmittance at different temperatures and times on June 5th in Nanjing: (A) 6:00 AM and (B) 12:00 PM. (C) Transmittance of SL-PNIPAm-based smart window at different thickness. (D) Transmittance of SLW at 300 to 2,500 nm at different temperature. (E) Transmittance of SLW from 300 to 900 nm. (F) Transmittance of SL-PNIPAm-based smart window at different temperatures near the LCST. (G) Comparison of *T*_lum_ and Δ*T*_solar_ values for SL-PNIPAm-based smart window and other works.

SL-PNIPAm solution with high luminous transmittance (*T*_lum_) and adjustable LCST is poured between the prepared double-layer hollow quartz glass to manufacture SLW (Fig. S6). Figure 3A illustrates the transparent state of SLW of 50 × 50 cm^2^ at 6 o’clock in the morning on June 6 in Nanjing (China), exhibiting extremely high sunlight transmittance and providing clear visibility through the window from the opposite side. At 12 o’clock, as the temperature rose, SLW underwent a rapid phase transition from a transparent to an opaque state, resulting in a loss of field of view and demonstrating a switchable optical property. Moreover, after manual touch or heat treatment, SLW rapidly undergoes a phase transition and becomes opaque, demonstrating superior response speed (Fig. 3B). As shown in Fig. 3C, the space thickness had almost negligible effect on the transmittance in the visible band, while the transmittance in the infrared band decreased with the increase of the thickness. In the different thicknesses of SLW, the 1-mm thickness completely blocks sunlight at high temperatures and has the highest transmittance at low temperatures, which shows the best luminous transmittance (*T*_lum_ = 96.8%) and solar modulation efficiency (Δ*T*_solar_ = 89.7%). SLW of 1-mm thickness is used in subsequent tests.

We further showcased the thermochromic performance of SL-PNIPAm by comparing its solar modulation capability and luminous transmittance with those of pristine PNIPAm. Figure 3D illustrates the transmittance of an SLW across the entire solar spectrum. At a temperature of 20 °C, SL-PNIPAm exists in a liquid state, and SLW exhibits the same high sunlight transmittance as pristine PNIPAm. Notably, the 2 declines in transmittance at 1,430 and 1,930 nm are due to the water molecule vibration [40]. At a temperature of 40 °C, our SLW effectively blocks all sunlight wavelengths, preventing solar heat from entering the windows. In contrast, pristine PNIPAm hydrogel fails to entirely block near-infrared (NIR) rays at elevated temperatures, leading to increased heat entering the room and higher energy consumption for air conditioning cooling. The transmittance of SLW closely resembled that of PNIPAm smart window (PSW) in visible and infrared wavelengths, excluding the ultraviolet (UV) range. A marked decline in transmittance was observed from the visible range to the UV range (Fig. 3E), providing SLW with excellent anti-UV properties. To systematically investigate transmittance changes at different temperatures, we employed a UV-Vis-NIR spectrophotometer at various temperature points (Fig. 3F). At temperatures below 28 °C, SLW maintains a high light transmittance, which gradually decreases as the temperature increases and becomes completely opaque at 32 °C. In Fig. 3G, we compare the sunlight regulation capabilities of our system to recent smart window studies, including hydrogels [24,26,30,31,33,41–44], ionic liquid gels [19,45,46], perovskite [47], and metal oxides [6,48–51]. Our system exhibits superior Δ*T*_solar_ and *T*_lum_ (Table S1). This capability enables effective temperature management within the room, allowing it to efficiently absorb solar heat at low temperatures and effectively block solar heat at high temperatures, thereby reducing energy consumption associated with indoor temperature regulation.

Figure S7A illustrates the reflectance of SLW and PSW at 40 °C. SLW exhibits an average reflectance of 85.9%, remarkably surpassing that of pristine PNIPAm hydrogel since the aperture-induced sunlight scattering of SL-PNIPAm enables superior solar heat reflection. High reflectivity in the solar spectrum minimizes solar heat absorption, thus reducing indoor air conditioning energy consumption in summer. We measured the thermal conductivity of SL-PNIPAm and compared it with that of pristine PNIPAm hydrogel and deionized (DI) water (Fig. S7B), revealing that SL-PNIPAm has a lower thermal conductivity than both pristine PNIPAm hydrogel and DI water. Low thermal conductivity minimizes the impact of the external environment on indoor temperature, thereby lowering energy consumption for indoor temperature regulation. Figure S7C illustrates the specific heat capacity (*C_p_*) of SL-PNIPAm and DI water. As the large *C_p_* of water is mainly attributed to the presence of hydrogen bonds, SL-PNIPAm exhibits a higher *C_p_* than water due to the introduction of additional functional groups (amide group and -C═O bond), which will generate more hydrogen bonds [52,53]. The high *C_p_* of SL-PNIPAm enables it to maintain a stable temperature while undergoing substantial temperature changes.

**Fig. 4.**
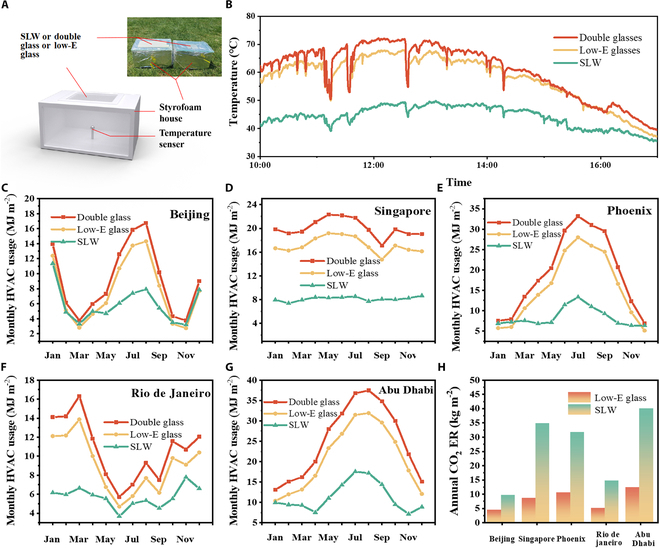
The energy-saving performance of SLW. (A) Device diagram of indoor simulation experiment. (B) Real-time temperature evolution curves of the simulation setup for SLW, low-E glass, and double-glazed glass. Monthly energy consumption of SLW, low-E glass, and double-layer glass window in the climate condition of (C) Beijing, (D) Singapore, (E) Phoenix, (F) Rio de Janeiro, and (G) Abu Dhabi. (H) Annual CO_2_ reduction for SLW and low-E glass compared to standard double glass.

The indoor simulation experiments were conducted to study the actual cooling effect of SLW. We constructed a foam device (Fig. 4A) to simulate the indoor environment, featuring an 8 × 8 cm^2^ opening above the closure device equipped with an SLW, low-E glass window, or a double-layer glass window, and a temperature sensor positioned 5 cm below each window. To mitigate the impact of heat conduction on the experimental results, we constructed the experimental device using a foam box with low thermal conductivity. Additionally, the foam box was covered with a layer of aluminum foil to reduce the effects of sunlight (Fig. S8). Figure 4B illustrates the temperature changes in foam device with the SLW, low-E glass window, and double-layer glass window (10:00 AM to 5:00 PM, September 8th in Nanjing China). As the sunlight intensity increased, the temperature of all 3 entities rose, but SLW exhibited the smallest increase. At maximum solar illumination, the temperature in foam device with SLW decreases by 22 °C compared to the double-layer glass window, while with the low-E glass window, its temperature drops by 18 °C. During the indoor simulation test, temperature fluctuations occurred several times, likely due to drifting clouds blocking the sun. SLW demonstrates superior temperature regulation ability in hot weather, effectively fulfilling the thermal adjustment functions required for energy-efficient buildings.

To assess the energy-saving capabilities of SLW in practical building design, we conducted energy-saving simulations using the actual building dimensions within the EnergyPlus software. The building measures 9 m in length, 6 m in width, and 3 m in height (Fig. S9). We further employed weather data from Beijing, Singapore, Phoenix, Rio de Janeiro, and Abu Dhabi, from different continents, facilitating a comprehensive study of SLW smart windows’ capability for all-weather temperature regulation and energy saving, thereby identifying their energy-saving potential. We also compared the results with standard double glass and low-E glass. Monthly HVAC energy consumption for all 3 window types in these areas is illustrated in Fig. 4C to G, respectively. Notably, during the period from April to September, SLW demonstrated superior energy efficiency compared to both standard double glass and low-E windows across all 5 cities. In Singapore, Rio de Janeiro, and Abu Dhabi, SLW consistently exhibited lower monthly consumption than low-E glass and standard glass. Particularly in Abu Dhabi, which represents a hot climate, SLW achieved a remarkable 54.5% annual HVAC energy savings compared to standard glass (in contrast to low-E glass’s 16.7% savings; Fig. S10). Additionally, we computed the carbon ER attributable to SLW and low-E glass relative to standard double glass of these 5 cities (Fig. 4H), and in Abu Dhabi, SLW achieved an annual carbon ER of 40 kg m^−2^, while low-E glass yielded only 12.2 kg m^−2^. These simulation results underscore SLW’s promising performance in conserving energy and reducing emissions across multiple urban contexts.

**Fig. 5.**
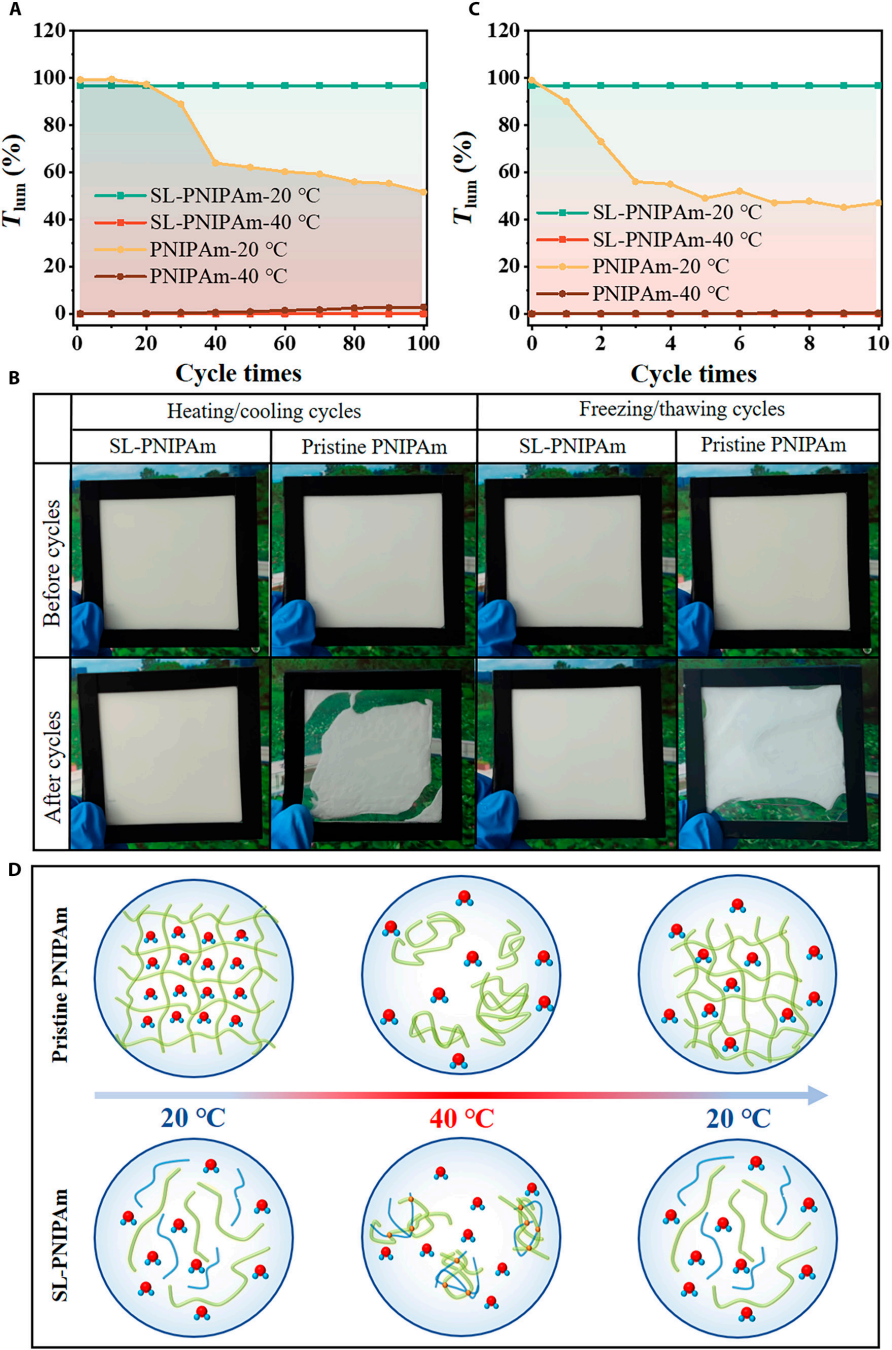
Cyclic stability testing and failure mechanism illustration. (A) Value of *T*_lum_ at 20/40 °C of SL-PNIPAm and pristine PNIPAm hydrogel after 100 heating/cooling cycles. (B) Photos of SL-PNIPAm and pristine PNIPAm after 100 heating/cooling cycles and 10 freezing/thawing cycles. (C) Value of *T*_lum_ of SL-PNIPAm and pristine PNIPAm hydrogel after 10 freezing/thawing cycles. The freezing temperature is set to −30 ° C. (D) Schematic illustration of SL-PNIPAm and pristine PNIPAm hydrogel after thermochromic cycles. SL-PNIPAm essentially maintains its structure, whereas pristine PNIPAm hydrogel undergoes irreversible volume shrinkage, leading to the thermochromic performance degradation. Green, PNIPAm molecular chain; blue, AMEO molecular chain; orange, cross-linking point between the 2 molecular chains.

The practical applications of the designed smart windows require careful consideration of their cyclic stability and performance under extreme conditions. We further evaluate the cyclic stability of SL-PNIPAm by measuring the luminous transmittance after subjecting it up to 100 heating/cooling cycles. Figure 5A illustrates that *T*_lum_ of PSW decreases markedly and drops to less than 50% after 100 heating/cooling (20/40 °C) cycles. In contrast, SLW maintains its initial level of transmittance after 100 heating/cooling cycles at both 20 and 40 °C. The change of Δ*T*_solar_ across heating/cooling cycles is depicted in Fig. S11. Comparable to *T*_lum_, Δ*T*_solar_ remains essentially unchanged in SLW throughout the cycles. However, in PSW, Δ*T*_solar_ exhibits a decreasing trend as the number of cycles increases. Figure 5B depicts the photos of PSW after 100 heating/cooling cycles, while the transmittance of PSW remains relatively stable at high temperatures. It experiences volume contraction and develops an uneven surface during cycling, resulting in a considerable degradation of the thermochromic performance. In 100 heating/cooling cycles, the states PSW were recorded by photos at intervals of 20 times during the heating/cooling cycles (Fig. S12). PSW exhibits surface nonuniformity at 20 cycles and starts to shrink at 40 cycles, whereas SLW maintains a consistently high *T*_lum_ and a flat surface throughout the cycling. As the temperature rises, the crosslinking degree of the 3D network of PNIPAm increases [34], enhancing its structural stability, impeding the complete restoration of the 3D network to its fully expanded state, resulting in the observed contraction phenomenon following repeated cycles of heating and cooling (Fig. 5C). The formation and breaking of dynamic imine bonds enable SL-PNIPAm to transition between the liquid and solid states. In the liquid state, SL-PNIPAm lacks a 3D cross-linked NIPAm network; thereby, the initial state can be restored at low temperatures. Additionally, the fluidity of the liquid state enhances the smart window’s self-repair capability, contributing to its excellent cyclic stability. Moreover, SL-PNIPAm did not produce shrinkage even when it was exposed to high temperature for a long time (Fig. S13).

While most thermochromic smart windows are optimized for hot days, they often exhibit inadequate performance in cold environments. The hydrogel thermochromic smart window contains a considerable amount of water, which can freeze in low temperatures during winter, leading to shrinkage like heating/cooling cycles. SLW can maintain high *T*_lum_ without any degradation even after undergoing numerous freezing/thawing (−30/20 °C) cycles. Figure 5D illustrates *T*_lum_ of both SL-PNIPAm and pristine PNIPAm hydrogel after 10 freezing/thawing cycles at 20 and 40 °C, respectively. Similar to the heating/cooling cycles, *T*_lum_ of PSW also exhibited a marked decrease. During the freezing/thawing cycles, Δ*T*_solar_ of SLW remains consistent, whereas Δ*T*_solar_ of PSW experiences a notable decrease, potentially impeding the normal functionality of the window (Fig. S14). After 10 freezing/thawing cycles, pristine PNIPAm hydrogel undergoes more pronounced contractions (Fig. 5B and Fig. S15). In contrast, SL-PNIPAm still maintains structural integrity.

After subjecting both SL-PNIPAm and pristine PNIPAm to heating/cooling or freezing/thawing cycles, we freeze-dried them and examined the alterations in their pore structures using SEM. These observations were subsequently compared with their pre-cycling states, as depicted in Fig. S16. Notably, the pore size of pristine PNIPAm underwent a substantial reduction following the cycles, attributable to its shrinkage. Conversely, SL-PNIPAm exhibited minimal changes in pore size, effectively preserving its original structure throughout the process. To assess the service life of SLW, we conducted a calculation utilizing the Hallberg–Peck model [54], derived from the Arrhenius equation. The expression for this model is as follows:

$$\mathrm{AF}=\exp\left[\frac{E_{\alpha }}{k}\times \left(\frac{1}{T_{\mathrm{use}}}-\frac{1}{T_{\text{test}}}\right)\right]\times {\left(\frac{{\mathrm{RH}}_{\text{test}}}{{\mathrm{RH}}_{\mathrm{use}}}\right)}^{n}$$ (1)

Here, *AF* represents the acceleration factor and *E_α_* (in 0.3 to 1.2 eV) denotes the activation energy of the chemical reaction. The constant *k* represents the Boltzmann constant, with a value of 8.617 × 10^−5^ eV K^−1^. *T*_use_ and *T*_test_ represent the operating temperature and the accelerated test temperature, respectively, both measured in kelvin. *RH*_use_ and *RH*_test_ correspond to the operating humidity and the accelerated test humidity, respectively. For the accelerated aging process, SLW was placed at 80 °C. Since SL-PNIPAm is enclosed, the impact of humidity on its lifespan is negligible. Assuming an *E_α_* value of 1.0 eV, we obtained an acceleration factor of *AF* = 429.2. After exposure to 80 °C for 4 days, SLW exhibited noticeable shrinkage, indicating its failure, as depicted in Fig. S17A. Based on these observations, we estimated its lifespan as *T* = 4 × 429.2/365 ≈ 4.7 years. However, it is noteworthy that at low temperatures, SL-PNIPAm exists in a liquid state and possesses self-healing properties. When the accelerated aging SLW was allowed to rest at room temperature for 48 h, it returned to its original state, as shown in Fig. S17B. Therefore, the actual lifespan of SLW is much longer than the theoretical estimate.

## Conclusion

In summary, we present a facile approach to endow PNIPAm with a solid–liquid transition property by incorporating a silane coupling agent (AMEO). The fluid nature of the liquid enables SL-PNIPAm to change its transmittance within 5 s according to ambient temperatures. SL-PNIPAm can be easily encapsulated within 2 glass panels to prepare SLW, which exhibits an exceptionally high luminous transmittance (*T*_lum_ = 96.8%) and solar modulation (Δ*T*_solar_ = 89.7%). Remarkably, the indoor temperature with SLW had an 18 °C reduction compared to low-E glass. SLW demonstrates excellent energy efficiency in different cities on 3 continents, saving up to 54% of HVAC energy and reducing annual CO_2_ emissions by 40 kg m^−2^ compared to double-layer glass window. Importantly, SL-PNIPAm exhibits superior cyclic stability during heating/cooling (freezing/thawing) cycles. This work provides a new direction for the design of hydrogel-based thermochromic smart windows and demonstrates substantial importance to energy conservation and emission reduction.

## Materials and Methods

### Materials

NIPAm (99.9%) was purchased from TCI, ammonium persulfate (APS; 99.99%), *N*,*N*′-methylenebis (acrylamide) (MBAA; 99%), *N*,*N*,*N*′,*N*′-tetramethylethylenediamine (TEMEDA; 99%), deuterium (D_2_O; 99.9%), and AMEO (99.8%) were purchased from Aladdin (Shanghai, China) and used without further purification. DI water was used for all experiments.

### Fabrication of SL-PNIPAm and SLW

NIPAm (1.05 g) was dissolved in DI water (10 ml), stirring until the solution became transparent. Argon gas was injected into the solution for 30 min to remove oxygen in the solution, and the solution was put in an ice bath for 10 min. After that, 0.01 g of APS (initiator), 0.5 ml of AMEO, and 0.01 ml of TEMED (catalyst) were added, stirring until the solution was evenly mixed, and the reaction was carried out at room temperature for 10 h to obtain SL-PNIPAm. SLW can be obtained by pouring the precursor solution or liquid SL-PNIPAm into the prepared mold.

### Characterization

UV-Vis-NIR transmittance was performed on a Shimadzu UV 3600 with a wavelength from 200 to 2,500 nm. During the test, the sample is packed in a prepared glass mold with different thicknesses, and the temperature control module is external. The FT-IR spectra of SL-PNIPAm and pristine PNIPAm were tested with the attenuated total reflectance (ATR) module equipped with the device (Thermo Fisher Scientific Nicolet iS10). Before the test, samples were heated to the set temperature and waited for 10 min. The Bruker Avance III HD 500MHz was used to measure the hydrogen spectrum of SL-PNIPAm at different temperatures. SEM was performed on a Nova Nano SEM450 with a 30-kV working voltage. For the SEM sample preparation, samples in the opaque and transparent states were immersed in liquid nitrogen immediately to maintain the microstructure, followed by freeze-drying in the freeze-dryer (SCIENTZ-12N, China) for 72 h. The responsive temperature of the simulated indoor experiment was recorded by TP1708 (TOPRIE, China). The rheological behaviors of SL-PNIPAm were analyzed by Haake Mars60 (Germany) with a 25-mm plane plate. Temperature sweeping experiments were conducted with a strain amplitude of 0.5% and a fixed frequency of 1 Hz. The differential scanning calorimetry curves of SL-PNIPAm with different AMEO content were measured by NETZACH DSC214, and the LCST was determined. The thermal conductivity and specific heat capacity of SL-PNIPAm were measured by a flash thermal conductivity meter (NETZACH LFA467). The XPS spectrum was tested by Thermo Fisher Scientific K-Alpha. The weight average molecular weight of SL-PNIPAm was tested by Agilent PL-GPC220. The transmittance of the glass panels was used as the baseline during the measurement. *T*_lum_ and *T*_solar_ was calculated by Eq. 2 [55]:

$$T_{\mathrm{lum}/\text{solar}}=\frac{\int T\left(\lambda \right)\varphi_{\mathrm{lum}/\text{solar}}\left(\lambda \right)d\lambda }{\int \varphi_{\mathrm{lum}/\text{solar}}\left(\lambda \right)d\lambda }$$ (2)

where T(λ) denotes spectral transmittance, *φ*_lum_(λ)is the standard luminous efficiency function of photopic vision in the wavelength range of 380 to 780 nm [26], and *φ*_solar_(λ) is the solar irradiance spectra for air mass 1.5 (corresponding to the sun standing 37° above the horizon with 1.5-atm thickness, corresponding to a solar zenith angle of 48.2°), respectively [26]. Δ*T*_solar_ was calculated by Eq. 3:

$$∆T_{\text{solar}}=T_{\text{solar},20{}^{\circ}C}-T_{\text{solar},40{}^{\circ}C}$$ (3)

### Model construction

Two types of models were built, that is, the ones at 20 and 40 °C. For the models at 20 °C, 2 AMEO molecules and 12 PNIPAm chains with *n* = 4 (Fig. S18) were randomly placed into the simulated box with a size of 35 Å × 35 Å × 35 Å and periodic boundary. The initial distance among these polymer molecules was larger than 10 Å to model uniform distribution. Then, the grand canonical Monte Carlo (GCMC) method was utilized to adsorb water molecules into the box to achieve a solution environment. This process proceeded 300 million steps to reach 1 g/m^3^ of water density. Concerning the model at 40 °C, above PNIPAm chains were connected based on AMEO by imine bond formation (Fig. S19). The connected number was from 1 to 6 for each AMEO molecule, which corresponds to 6 models constructed at 40 °C to investigate the effect of polymerized degree. Finally, water addition by GCMC was conducted as the above procedure.

### Force field and simulation procedure

Consistent valence force field (CVFF) was conducted to model this polymer system. It was extensively utilized in the simulation of the structure and dynamics of peptides, proteins, and other polymer systems and has been confirmed to be accurate to simulate polymer with low molecular weight (e.g., carboxylic acids and amides) [56–58]. Previously published papers confirmed that the bond lengths, bond angles, torsion angles, and dihedral of carbon chains of polymer chains can be accurately described under CVFF [59]. The simulation was performed on large-scale atomic/molecular massively parallel simulator (LAMMPS). Nose–Hoover thermostat and Verlet algorithm were applied in the simulation. Time step was set as 1 fs. Energy minimization was performed to optimize initial structure [60]. Then, NPT (constant number of particles, pressure, and temperature) run was conducted at 1 atm and 300 K/320 K for 500 ps. Next, NVT (constant number of particles, volume, and temperature) run was followed, which was performed at 300 K/320 K for 500 ps to obtain a well-equilibrated molecular structure.

### Energy simulation

A model house measuring 9 m by 6 m by 3 m, featuring 4 windows positioned centrally on each of the 4 walls (refer to Fig. S9), was simulated using EnergyPlus software. Separate simulations were conducted for double glass, low-E glass, and SLW, with pertinent optical data delineated in Table S2 for each window type. The window performance was evaluated across various geographical locations, including Beijing, Singapore, Phoenix, Rio de Janeiro, and Abu Dhabi, to account for diverse climatic conditions. Meteorological data utilized for the simulations were sourced from the official EnergyPlus website. Notably, the thermal regulation of SLW was assessed based solely on outdoor temperature, independent of indoor conditions. Furthermore, an energy conservation analysis was conducted by integrating an HVAC system calibrated to maintain a constant temperature of 26 °C, with the resulting energy consumption for heating and cooling recorded for each model. Annual HVAC usage (*E*) is the result of adding the energy consumption of each month, and annual energy saving (*E*_S_) was calculated by Eq. 4:

$$E_{s,\mathrm{Low}-E/\mathrm{SLW}}=E_{\text{Double}}-E_{\mathrm{Low}-E/\mathrm{SLW}}$$ (4)

Annual carbon ER was calculated by Eq. 5 in carbon emission factor method:

$${\mathrm{ER}}_{s,\mathrm{Low}-E/\mathrm{SLW}}=E_{s,\mathrm{Low}-E/\mathrm{SLW}}\times 0.785/3.6$$ (5)

where 0.785 kg/kWh is the mass of CO_2_ produced by 1 kWh of electricity consumed and 3.6 is the conversion relationship between J and kWh.

## Data Availability

The authors confirm that the data supporting the findings of this study are available within the article and its Supplementary Materials.

## Supplementary Materials

Supplementary 1 Figs. S1 to S19 Tables S1 and S2 Movie S1 References
