# Supplementary material for: Thermochromic Smart Windows with Ultra-High Solar Modulation and Ultra-Fast Responsive Speed Based on Solid–Liquid Switchable Hydrogels
Source: Research (Wash D C). 2024 Sep 3;7:0462. doi: 10.34133/research.0462 (PMC12327384; doi:10.34133/research.0462)
Supplement: Supplementary 1 — Figs. S1 to S19 Tables S1 and S2 Movie S1 References [file research.0462.f1.zip › Supporting Information.pdf]

Supporting Information

Thermochromic Smart Windows with Ultra-high Solar modulation and Ultra-fast Responsive Speed based on Solid-Liquid Switchable Hydrogels

Guangjun Zhu[1, 3†], Xu Gang[2, 3†], Yu Zhang[4], Guo Lu[1, 3], Xuan Cai[5], Wei Zhang[2, 3*], Wei She[1, 3*], Changwen Miao[1, 3]

[1]State Key Laboratory of High Performance Civil Engineering Materials, [2]Jiangsu Key Laboratory of Advanced Metallic Materials, [3]School of Materials Science and Engineering, Southeast University, Nanjing, 211189, China.

[4]School of Civil Engineering and Architecture, Shandong University of Science and Technology, Qingdao, 266590, China.

[5]Wuhan National Laboratory for Optoelectronics, School of Optical and Electronic Information, Huazhong University of Science and Technology, Wuhan, 430074, China.

†These authors contributed equally to this work.

*Corresponding authors: w69zhang@seu.edu.cn; weishe@seu.edu.cn

Supporting Figures

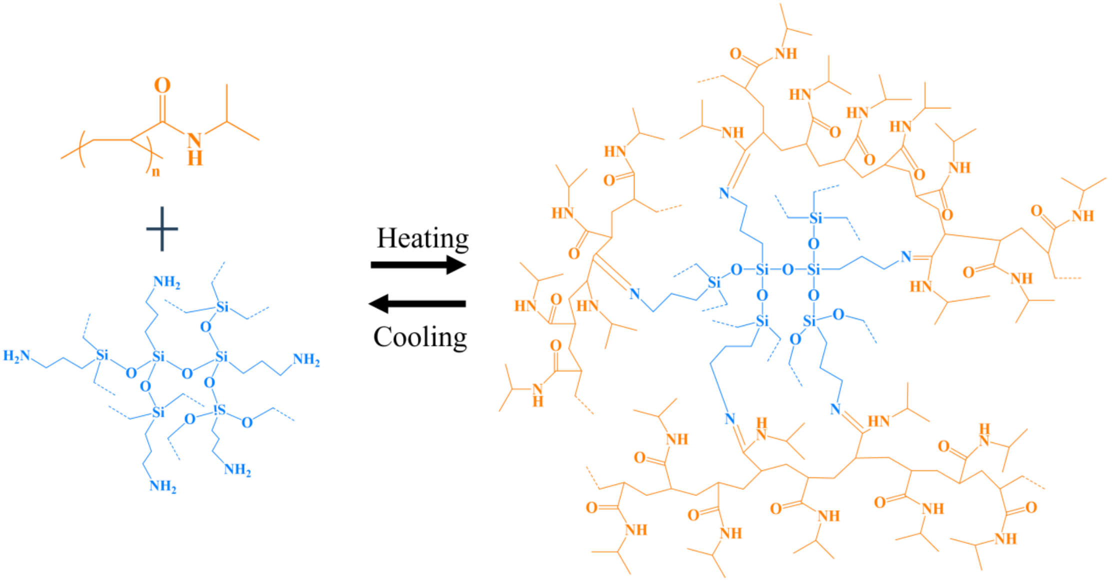

Fig. S1. At low temperatures, the PNIPAm fails to form a 3D network because MCA cannot form imine bond with the C=O bond of PNIPAm in water-rich environments,

resulting in a fluid liquid state of SL-PNIPAm. As the temperature rises, the molecular chains of PNIPAm undergo a transition from the expanded state to the contracted state and phase separation occur, which provides the ideal conditions for the formation of imine bond with the silanol network, resulting in a crosslinked 3D SL-PNIPAm network in a solid state, the dashed lines represent the undrawn chemical structures.

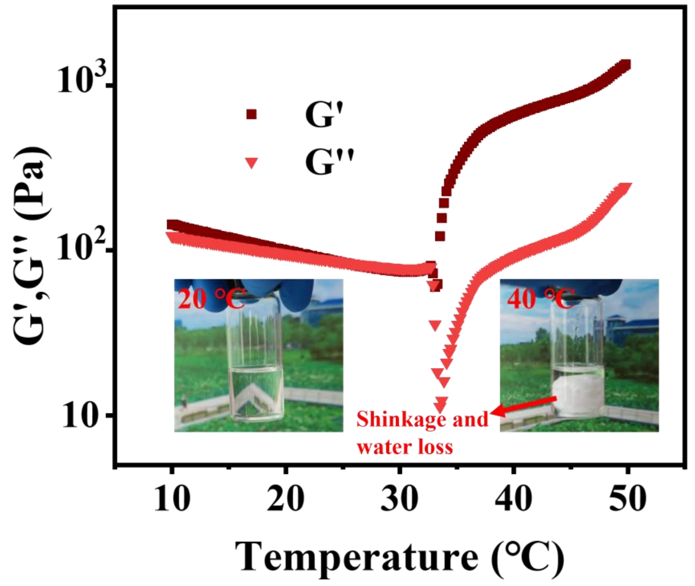

Fig. S2. Rheological properties of non-modified PNIPAm solution (without AMEO) at different temperatures.

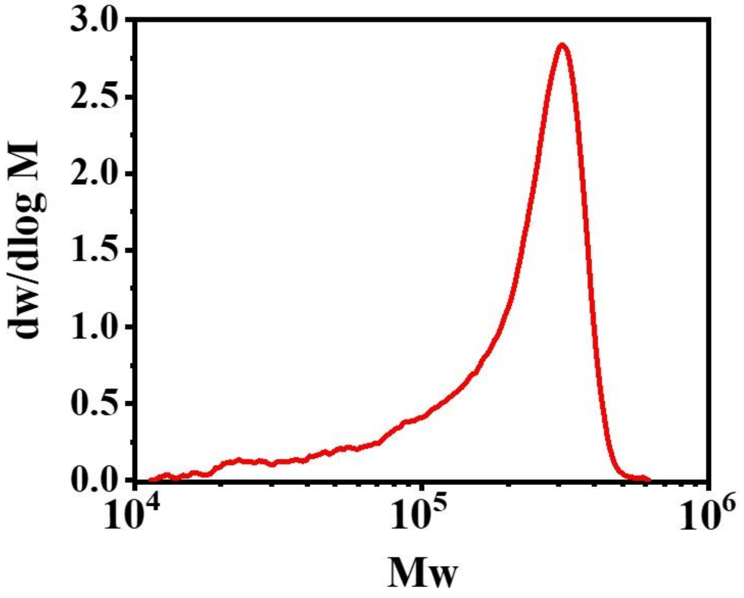

Fig. S3. Weight average molecular weight distribution of SL-PNIPAm.

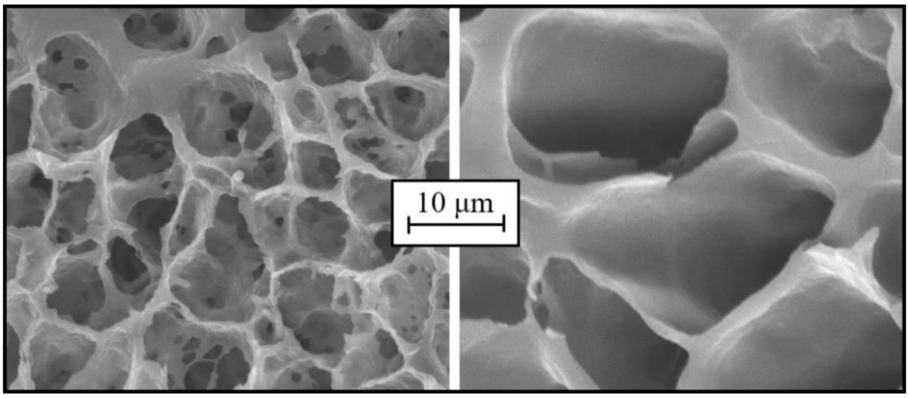

Fig. S4. Pristine PNIPAm at different temperatures (left, 20 °C; right, 40 °C) SEM photo after freeze-drying.

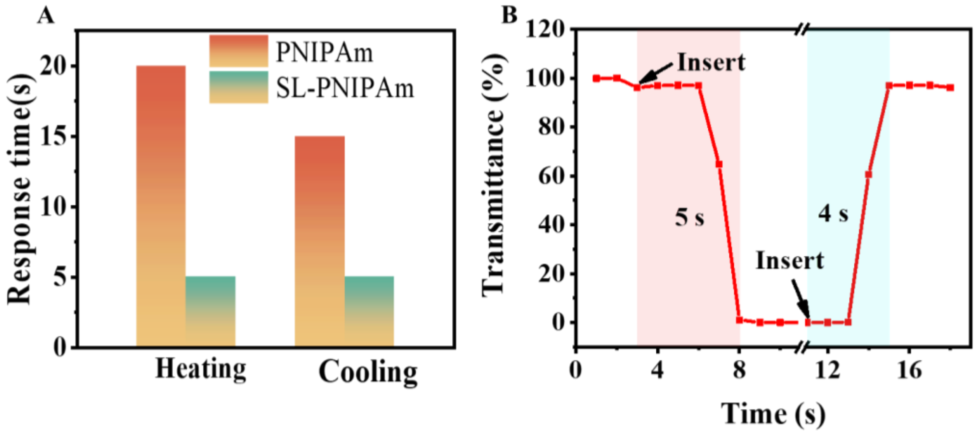

Fig. S5. (A) Response time of SL-PNIPAm and pristine PNIPAm during heating or cooling process. (B) The transmittance-time curve at 650 nm during the heating or cooling process.

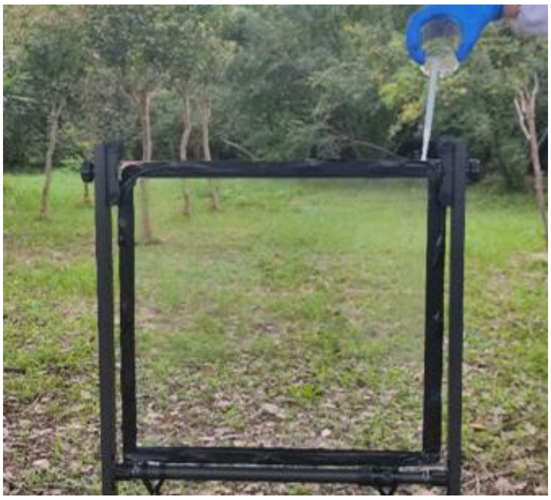

Fig. S6. SLW is prepared by pouring SL-PNIPAm precursor solution or liquid SL-PNIPAm into a 50 cm × 50 cm glass layer.

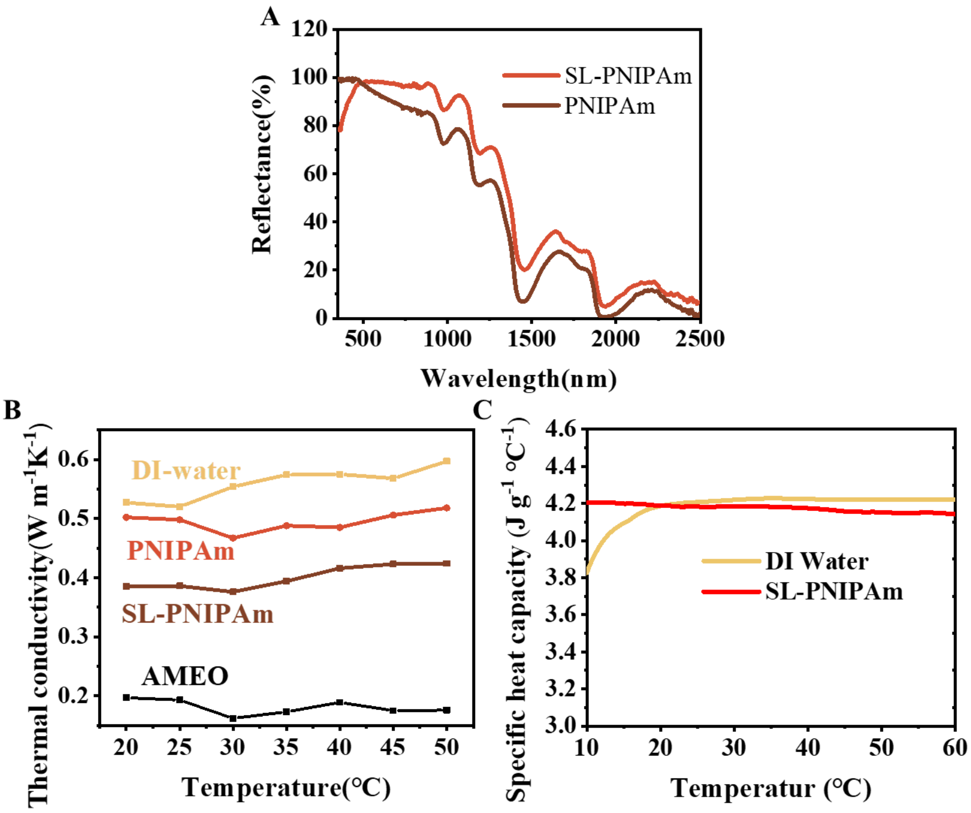

Fig. S7. (A) Reflectance of SL-PNIPAm and pristine PNIPAm. (B) Thermal conductivity of SL-PNIPAm, pristine PNIPAm and water. (C) Specific heat capacity of SL-PNIPAm.

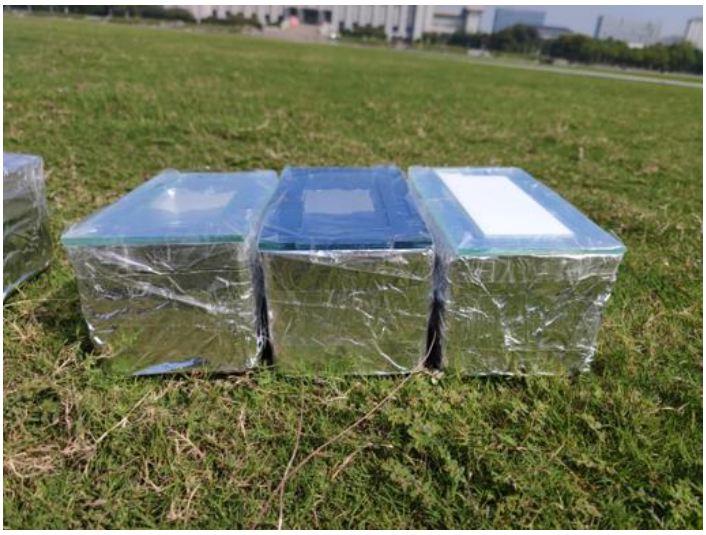

Fig. S8. A photo of a device that simulates laboratory experiments, with a layer of aluminum foil outside the foam box to reduce experimental errors.

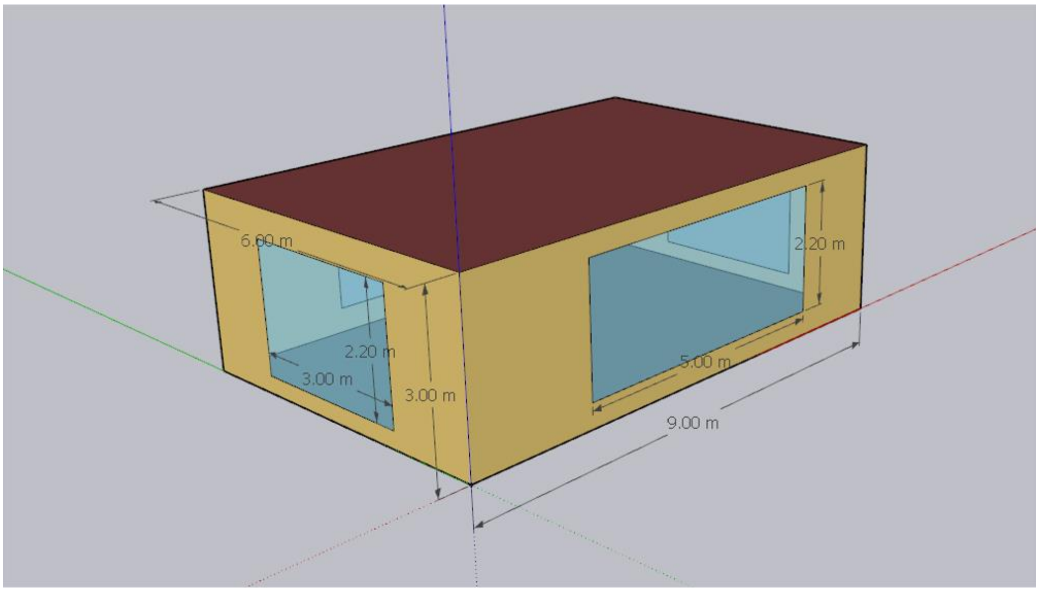

Fig. S9. Schematic diagram of the building model used in the principal simulation.

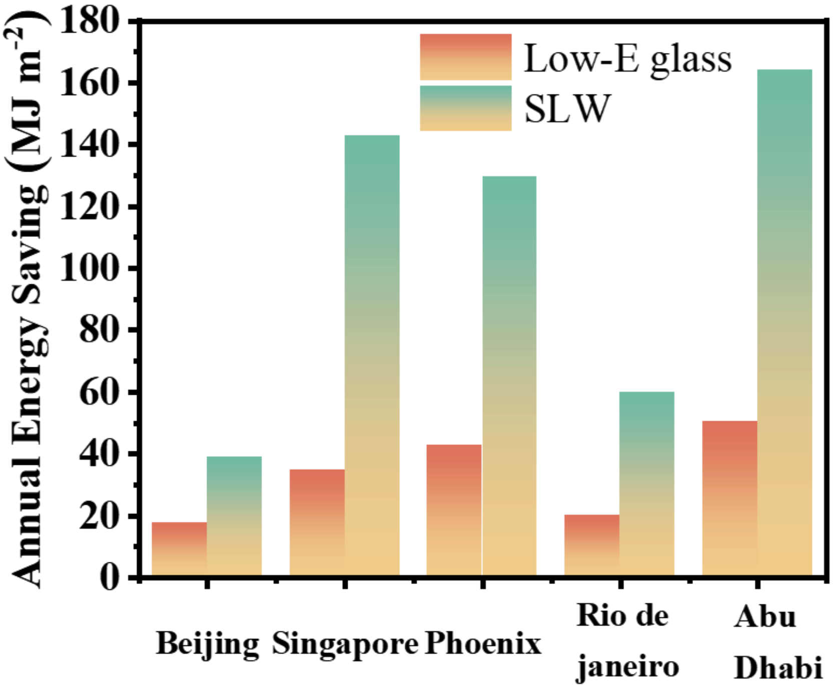

Fig. S10. Energy savings per year for Low-E glass and SLW in different cities relative to double glass.

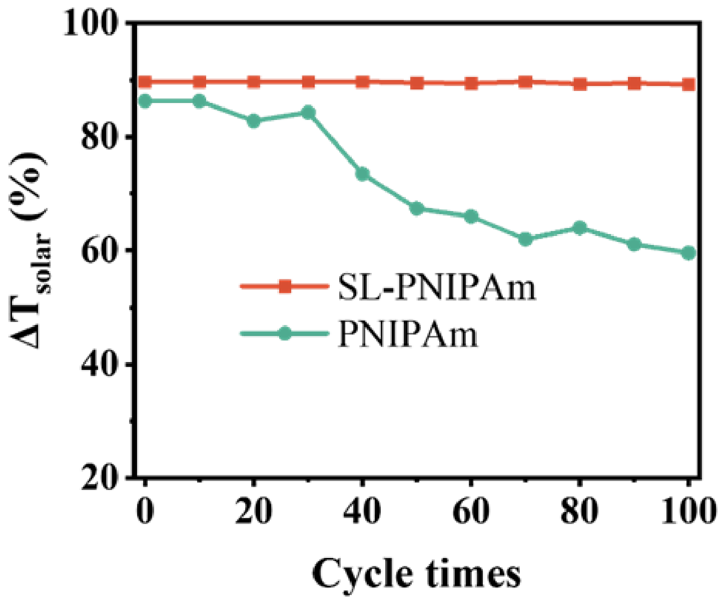

Fig. S11. The value of ΔT_{solar} of SL-PNIPAm and pristine PNIPAm hydrogel after undergoes 0 to 100 heating/cooling cycles.

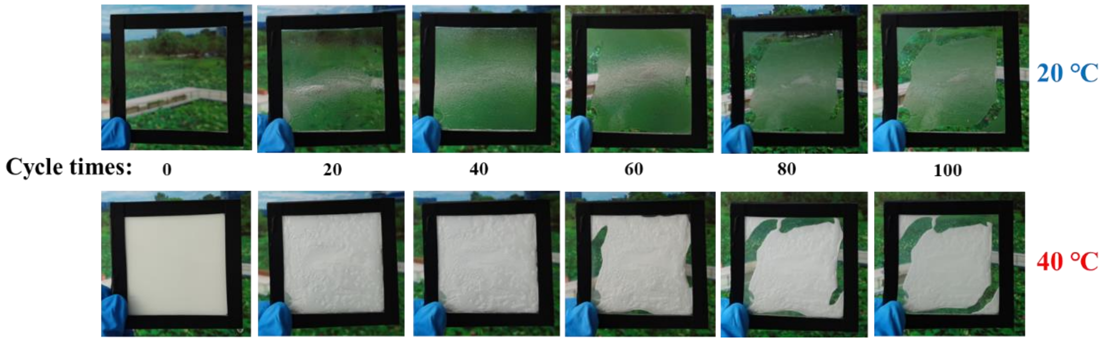

Fig. S12. Irreversible shrinkage of PNIPAm was observed in photographs after different heating/cooling cycles.

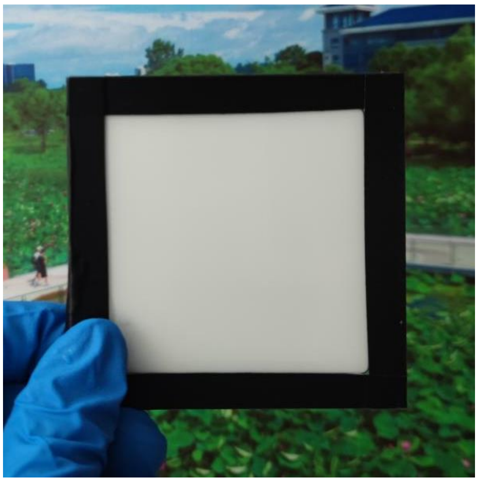

Fig. S13. Photos of SL-PNIPAm after storage at 40 °C for 24 hours.

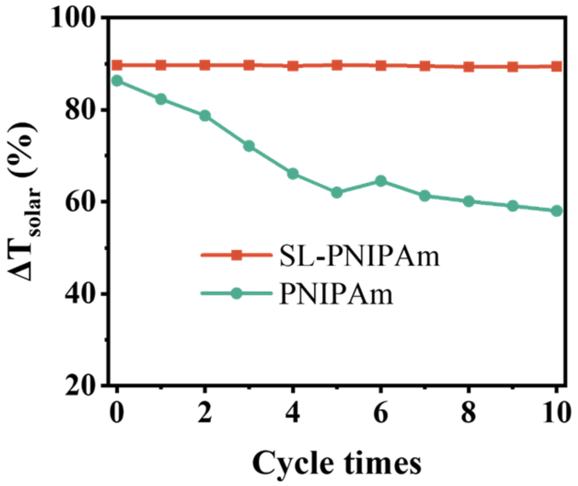

Fig. S14. The value of ΔT_{solar} of SL-PNIPAm and pristine PNIPAm hydrogel after undergoes 0 to 10 freezing/thawing cycles.

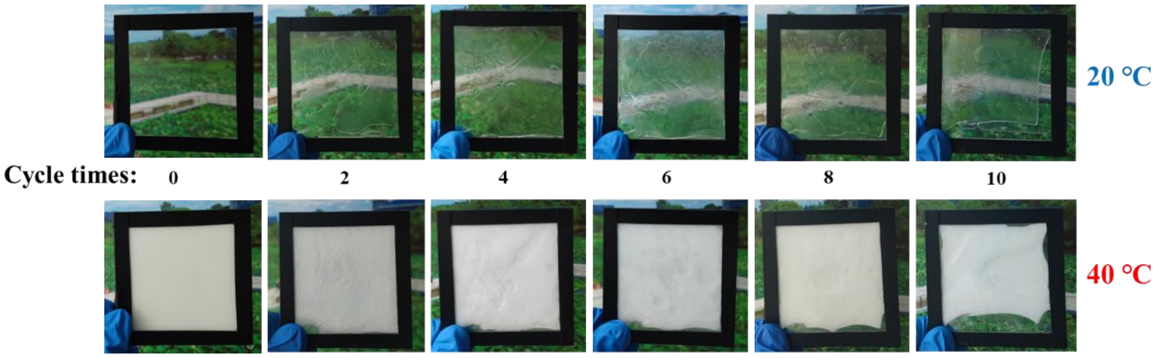

Fig. S15. Irreversible shrinkage of PNIPAm was observed in photographs after different freezing/thawing cycles.

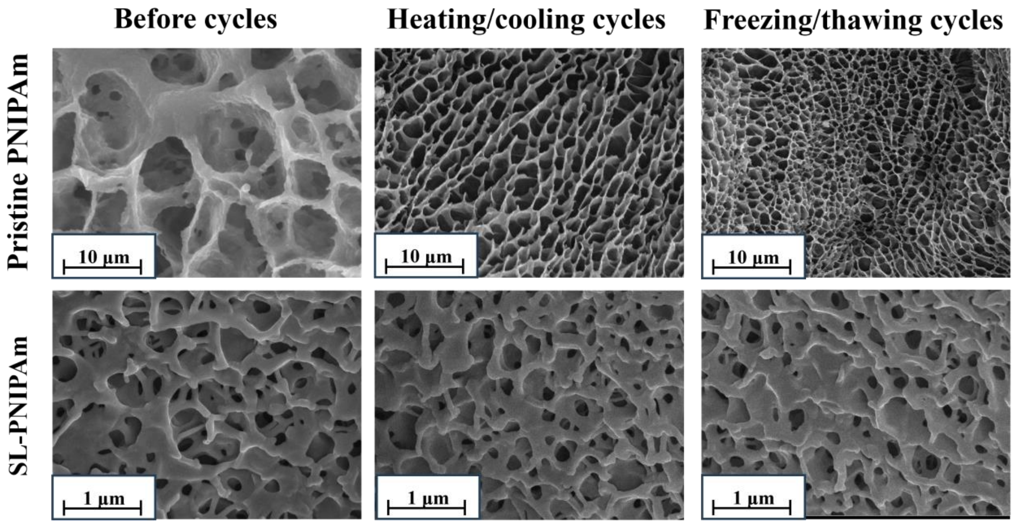

Fig. S16. SEM images of SL-PNIPAm and PNIPAm after heating/cooling or freezing/thawing cycles.

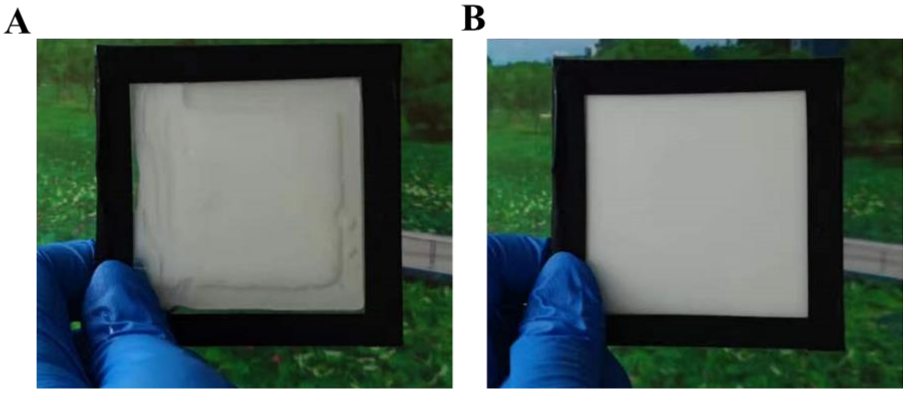

Fig. S17. (A) Accelerated aging SLW photos after four days. (B) Photos of SLW damaged by accelerated aging after being placed at room temperature for 48 h.

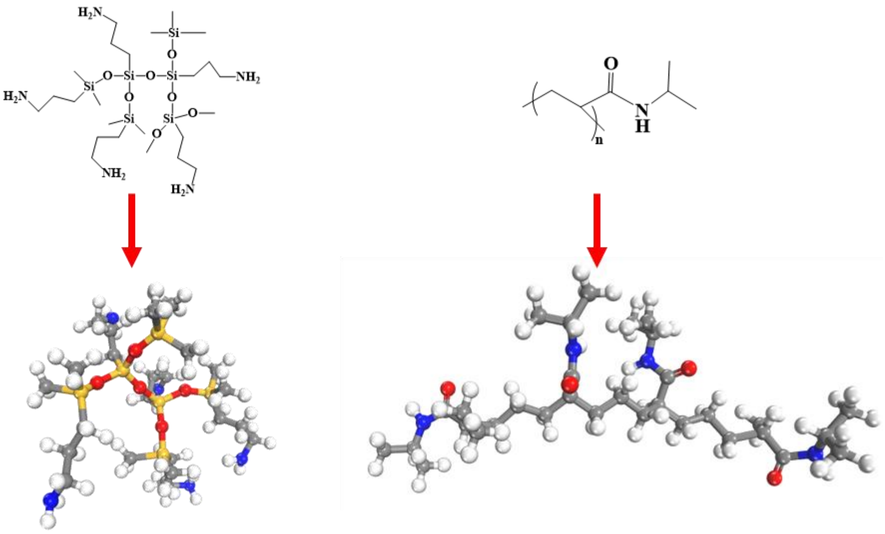

Fig. S18. AMEO molecule and PNIPAm chains.

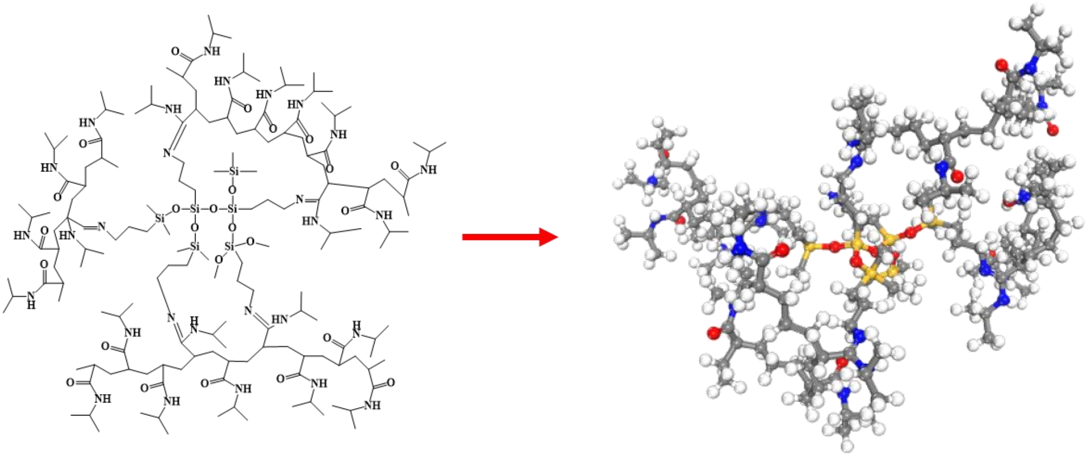

Fig. S19. PNIPAm chains were connected based on AMEO by imine bond formation.

Supporting Tables

Table S1 Comparison of thermochromic performance in various thermochromic smart windows utilizing different thermochromic materials.

Category	Materials	T_{lum} (%)	ΔT_{sol} (%)	Ref.
Hydrogels	Liquid PNIPAm	91.5	85.8	1
	POSS-poloxamer	98	66.9	2
	PNIPAm/AEMA	87.2	75.6	3
	PNIPAm/C_2H_5OH	89.89	71.81	4
	Glycerin/PNIPAm	90	60.8	5
	IPAm/HPC	80.7	64.5	6
	PNIPAm/AgNWs	78.3	58.4	7
	HPC/ITO	72	51	8
	Liquid PNIPAm	90	68.1	9
Ionogels	Ionic liquids/PU/ASCZ	95.1	83	10
	Cellulose/PAAm	85	49.8	11
	PNIPAm ionic gel	99	71	12
Perovskite	H-$MAPbI_{3-x}Cl_x$	86	23.7	13
VO_2	VO_2 film	41	14.9	14
	Rolled-up VO_2 SWs	61.01	42.14	15
	ITO/PMMA/VO_2	80	9	16
	W/VO_2 NWs	70	24.8	17
	ITO/VO_2/PVC	51.9	9.8	18
	**SL-PNIPAm**	**96.8**	**89.7**	**This work**

Table S2 Optical data of different windows.

	Double glass	Low-E glass	SLW (cold/hot)
Solar Transmittance	0.863	0.63	0.897/0
Emissivity (outer)	0.89	0.84	0.95
Emissivity (inner)	0.89	0.1	0.95

Supporting Movie

Movie S1. The SL-PNIPAm responds to temperature within 5 s.

Reference

1. Li J, Gu P, Pan H, Qiao Z, Wang J, Cao Y, Wang W, Yang Y. A Facile yet Versatile Strategy to Construct Liquid Hybrid Energy-Saving Windows for Strong Solar Modulation. *Adv Sci.* 2023;10(10):2206044.

2. Zhang Q, Jiang Y, Chen L, Chen W, Li J, Cai Y, Ma C, Xu W, Lu Y, Jia X, Bao Z. Ultra-Compliant and Tough Thermochromic Polymer for Self-Regulated Smart Windows. *Adv Funct Mater.*

2021;31(18):2100686.
3. Li X-H, Liu C, Feng S-P, Fang N. Broadband Light Management with Thermochromic Hydrogel Microparticles for Smart Windows. *Joule.* 2019;3(1):290-302.
4. Ding Y, Duan Y, Yang F, Xiong Y, Guo S. High-transmittance pNIPAm gel smart windows with lower response temperature and stronger solar regulation. *Chem Eng J.* 2023;460:Article 141572.
5. Li G, Chen J, Yan Z, Wang S, Ke Y, Luo W, Ma H, Guan J, Long Y. Physical crosslinked hydrogel-derived smart windows: anti-freezing and fast thermal responsive performance. *Mater Horiz.* 2023;10(6):2004-2012.
6. Feng Y, Wang S, Li Y, Ma W, Zhang G, Yang M, Li H, Yang Y, Long Y. Entanglement in Smart Hydrogels: Fast Response Time, Anti-Freezing and Anti-Drying. *Adv Funct Mater.* 2023;33(21):202211027.
7. Lin C, Hur J, Chao CYH, Liu G, Yao S, Li W, Huang B. All-weather thermochromic windows for synchronous solar and thermal radiation regulation. *Sci Adv.* 2022;8(17):eabn7359.
8. Wang S, Zhou Y, Jiang T, Yang R, Tan G, Long Y. Thermochromic smart windows with highly regulated radiative cooling and solar transmission. *Nano Energy.* 2021;89:Article 106440.
9. Zhou Y, Wang S, Peng J, Tan Y, Li C, Boey FYC, Long Y. Liquid Thermo-Responsive Smart Window Derived from Hydrogel. *Joule.* 2020;4(11):2458-2474.
10. Li B, Xu F, Guan T, Li Y, Sun J. Self-Adhesive Self-Healing Thermochromic Ionogels for Smart Windows with Excellent Environmental and Mechanical Stability, Solar Modulation, and Antifogging Capabilities. *Adv Mater.* 2023;35(20):202211456.
11. Chen S, Jiang G, Zhou J, Wang G, Zhu Y, Cheng W, Xu G, Zhao D, Yu H. Robust Solvatochromic Gels for Self-defensive Smart Windows. *Adv Funct Mater.* 2023;33(20):202214382.
12. Deng B, Zhu Y, Wang X, Zhu J, Liu M, Liu M, He Y, Zhu C, Zhang C, Meng H. An Ultrafast, Energy-Efficient Electrochromic and Thermochromic Device for Smart Windows. *Adv Mater.* 2023;35(35):202302685.
13. Liu S, Du Y. W, Tso CY, Lee HH, Cheng R, Feng S, Yu KM. Organic Hybrid Perovskite (MAPbI3-xClx) for Thermochromic Smart Window with Strong Optical Regulation Ability, Low Transition Temperature, and Narrow Hysteresis Width. *Adv Funct Mater.* 2021;31(26):2010426.
14. Zhao S, Shao Z, Huang A, Bao S, Luo H, Ji S, Jin P, Cao X. Dynamic full-color tunability of high-performance smart windows utilizing absorption-emission effect. *Nano Energy.* 2021;89:Article 106297.
15. Li X, Cao C, Liu C, He W, Wu K, Wang Y, Xu B, Tian Z, Song E, Cui J, Huang G, Zheng C, Di Z, Cao X, Mei Y. Self-rolling of vanadium dioxide nanomembranes for enhanced multi-level solar modulation. *Nat Commun.* 2022;13:7819.
16. Wang S, Jiang T, Meng Y, Yang R, Tan G, Long Y. Scalable thermochromic smart windows with passive radiative cooling regulation. *Science.* 2021;374 (6574):1501-1504.
17. Sheng S, Wang J, Zhao B, He Z, Feng X, Shang Q, Chen C, Pei G, Zhou J, Liu J, Yu S. Nanowire-based smart windows combining electro-and thermochromics for dynamic regulation of solar radiation. *Nat Commun.* 2023;14:3231.

18. Ke Y, Li Y, Wu L, Wang S, Yang R, Yin J, Tan G, Long Y. On-Demand Solar and Thermal Radiation Management Based on Switchable Interwoven Surfaces. *ACS Energy Lett.* 2022;7(5):1758-1763.
